# Shaping Precision Medicine: The Journey of Sequencing Technologies Across Human Solid Tumors

**DOI:** 10.3390/biomedicines13112660

**Published:** 2025-10-29

**Authors:** Wanwen Li, Chanyu Xiong, Chen Chu, Yun Zhang, Zihao Wang, Zunmin Wan, Peng Tang, Shikai Zhu, Yu Zhou

**Affiliations:** 1 Genetic Diseases Key Laboratory of Sichuan Province, Medical Genetics and Rare Diseases Center, Department of Laboratory Medicine, Sichuan Provincial People’s Hospital, School of Medicine, University of Electronic Science and Technology of China, Chengdu 610072, China; 18190867758@163.com (W.L.); chanyuxiong@foxmail.com (C.X.); zhangyunqqqqq@163.com (Y.Z.); wanzunmin19@mails.ucas.ac.cn (Z.W.);; 2 Department of Cancer Biology, Dana-Farber Cancer Institute, Boston, MA 02215, USA; chen_chu@dfci.harvard.edu; 3 Department of Genetics, Blavatnik Institute, Harvard Medical School, Boston, MA 02115, USA; 4 Sichuan Provincial Key Laboratory for Clinical Immunology Translational Medicine, Organ Transplant Center, Sichuan Provincial People’s Hospital, School of Medicine, University of Electronic Science and Technology of China, Chengdu 610072, China

**Keywords:** human solid tumors, genetic mutations, sequencing technology, clinical guidelines, precision medicine

## Abstract

Solid tumors collectively drive the global cancer burden, with profound molecular heterogeneity demanding precision and molecularly informed management. Advances in sequencing technologies have established molecular taxonomy as a cornerstone of clinical oncology, progressively superseding traditional histopathological classifications. Sanger sequencing remains the gold standard for validating guideline mandated actionable variants. Next-generation sequencing (NGS) has revolutionized early cancer detection through liquid biopsy applications and enabled the reclassification of diagnostically challenging tumor subtypes. Emerging long-read platforms offer unique capabilities to resolve complex genomic rearrangements, structural variants, and therapy-induced epigenetic remodeling. Consequently, therapeutic strategies are shifting from organ-centric approaches to mutation-specific interventions, exemplified by non-small-cell lung cancer, where molecular stratification drives substantial improvements in treatment response. Nevertheless, temporal tumor heterogeneity, biological contamination, and computational limitations highlight the urgent need for robust, integrated verification systems. Collectively, this evolution positions sequencing as the operational backbone of adaptive precision oncology across solid tumors. Here, we synthesize our laboratory findings with the current literature to comprehensively review the diagnostic, therapeutic, and prognostic applications of first- through fourth-generation sequencing technologies and discuss future directions in this rapidly evolving field.

## 1. Introduction

Solid tumors, originating from epithelial, mesenchymal, or specialized parenchymal cells, account for approximately 90% of newly diagnosed cancer cases worldwide [1]. According to the 2024 GLOBOCAN projections, the incidence of carcinomas, sarcomas, and gliomas is expected to rise steadily, further intensifying the global cancer burden [2]. The therapeutic paradigm for these malignancies has shifted from organ-centered approaches to molecularly guided strategies [3], recognizing that histopathological classifications fail to capture the clinically divergent behaviors of genomically distinct subgroups [4]. This shift is exemplified by the management of non-small-cell lung cancer (NSCLC): whereas platinum-based chemotherapy yields objective response rates (ORR) of only 20–35% in advanced disease [5], the discovery of *EGFR* mutations in 2004 and the subsequent development of targeted agents such as osimertinib have raised ORR to nearly 80% [6], demonstrating the transformative potential of molecular stratification. Collectively, these breakthroughs signal the shift from histology-based classifications to molecular taxonomy across the spectrum of solid tumor management.

Breakthroughs in sequencing technologies have been central to the development of these molecular frameworks, now incorporated into the WHO classification system [7]. The evolution of sequencing platforms reflects the stepwise resolution of key clinical challenges. Sanger sequencing first established reliable single-gene interrogation, but its low throughput limited utility in genomically heterogeneous tumors [8]. Next-generation sequencing (NGS) overcame scalability barriers through massively parallel sequencing, enabling simultaneous interrogation of hundreds of genes, but still struggled with complex structural variants, repetitive elements, and native epigenetic modifications. More recently, third-generation long-read platforms have addressed these limitations through single-molecule, real-time sequencing, offering comprehensive breakpoint resolution, methylation profiling, and transcriptome-wide variant detection at base-pair precision. The emergence of fourth-generation nanopore-based technologies further promises real-time sequencing at a lower cost, expanding accessibility for clinical use [8].

In this review, we synthesize current evidence on the clinical deployment of first- through fourth-generation sequencing technologies across the diagnosis, treatment, and prognosis of solid tumors. We further discuss platform-specific strengths and limitations, highlight our own laboratory findings, and provide a perspective on how these technologies collectively establish molecular profiling as a central driver of adaptive precision oncology.

## 2. Genetic Mutations in Human Solid Tumors

The therapeutic paradigm for human solid tumors has undergone a marked transition from histology-based classification to genomics-informed strategies, with comprehensive molecular profiling now positioned as a cornerstone of precision oncology [9]. Large-scale genomic studies have demonstrated that over 90% of malignant neoplasms harbor at least one clinically actionable driver alteration, underscoring the critical role of mutational analysis in guiding individualized care [10,11].

Pathogenic mutations are broadly categorized as somatic variants, which arise in specific tissues and drive tumor initiation or progression, and germline variants, which are present in all cells and confer hereditary cancer predisposition [10,12,13]. Approximately 5–10% of solid tumors are attributable to hereditary cancer syndromes such as Lynch syndrome [14], hereditary breast and ovarian cancer syndrome [15], and familial adenomatous polyposis [16,17]. For patients meeting National Comprehensive Cancer Network (NCCN) criteria—such as diagnosis before age 50 or the presence of multiple primary tumors—cascade genetic testing of first-degree relatives is recommended [18]. Early identification of pathogenic germline variants enables risk-reducing interventions (e.g., prophylactic surgery, enhanced surveillance) and facilitates targeted therapeutic selection, including *PARP* inhibitors for *BRCA1/2* mutation carriers [19].

**Figure 1:**
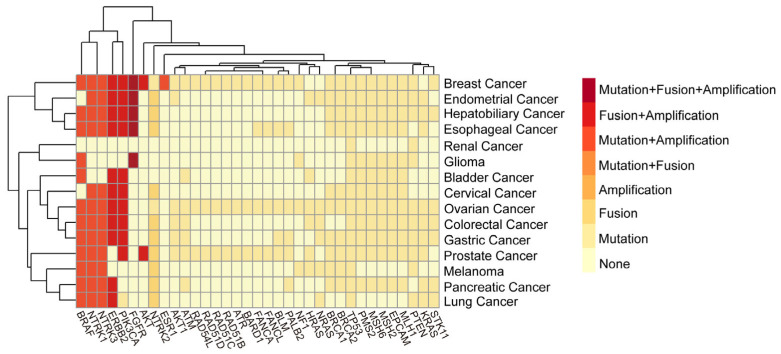
Mutation patterns of 35 commonly mutated genes across 15 solid tumors. The common mutation genes and mutation types of solid tumors from Sichuan Provincial People’s Hospital from January 2024 to January 2025 were analyzed and visualized in this figure. The figure summarizes the 6 possible mutation types of 35 gene mutations that are more common in the 15 solid tumors with the highest incidence rates.

Somatic driver mutations disrupt oncogenic homeostasis through three principal mechanisms: constitutive activation of proto-oncogenes, functional inactivation of tumor suppressor genes, and generation of oncogenic fusion proteins. Table 1 summarizes the most frequently mutated genes in the top 35 solid tumors based on the 2023 WHO classification. To supplement these global data, we collected the mutation data of 500 patients who underwent clinical genetic testing at Sichuan Provincial People’s Hospital from January 2024 to April 2025, for the purpose of analyzing the driving genes and mutation types. Testing was performed using the GENESEEQPRIME™ TMB, covering commonly mutated oncogenes and tumor suppressor genes across solid tumors. Library preparation, hybrid capture, and sequencing were conducted following the manufacturer’s protocols. Both tumor tissue and plasma (cfDNA) samples were included. Tissue samples were required to have ≥20% tumor cellularity. Minimum mean sequencing depth was set at ≥500× for tissue and ≥1000× for plasma. Variants were called using the vendor-provided bioinformatics pipeline with the following thresholds: minimum variant allele frequency (VAF) ≥ 2% for tissue and ≥0.5% for plasma. Only pathogenic or likely pathogenic variants were assessed. The most prevalent driver genes and mutation types are illustrated in Figure 1, providing a real-world snapshot of mutational landscapes across multiple tumor types. Furthermore, publicly available biomarker resources such as MyCancerGenome (https://www.mycancergenome.org/) currently catalog over 18,000 genetic biomarkers and more than 5500 genome-directed cancer therapies, reflecting the rapid expansion of genotype-driven treatment options. Collectively, the detection and interpretation of driver gene alterations have transformed cancer management from organ- and histology-based paradigms toward individualized treatment strategies, enabling more precise therapeutic planning and improved patient outcomes.

## 3. Sequencing Technology in Solid Tumor Diagnostics

**Figure 2:**
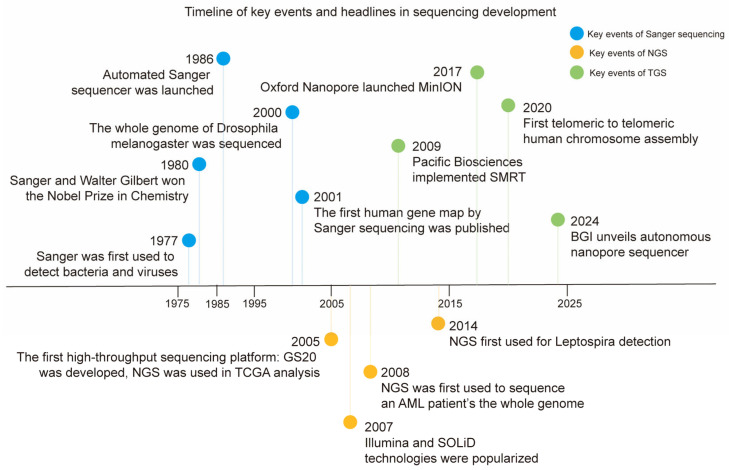
The chronological histories of Sanger sequencing, NGS, and TGS. Some milestones in the development of sequencing technologies (including Sanger sequencing, NGS, and TGS) are summarized in chronological order.

The continuous advancement of sequencing platforms has fundamentally transformed molecular profiling in solid tumors, shifting the diagnostic paradigm from histology-based classification to driver alteration-centric stratification. This progression is characterized by three generations of technologies, each with distinct mechanistic principles and clinical utilities. This part summarizes the chronological development history (Figure 2), common information, principles, and application of sequencing technologies.

### 3.1. First-Generation Sequencing (Sanger Sequencing)

**Figure 3:**
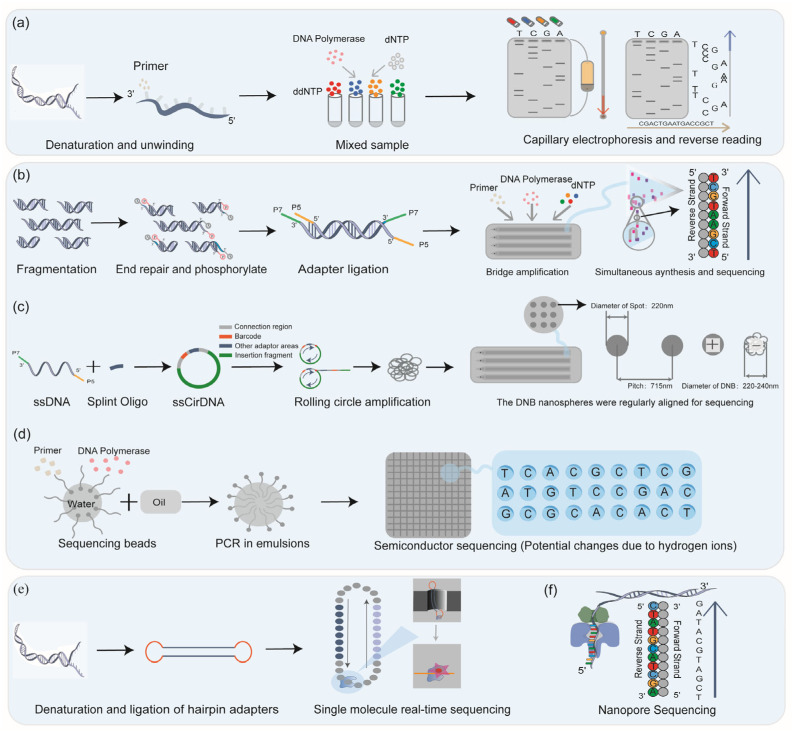
The principles of sequencing technology. Four different fluorescent ddNTPs are used for Sanger sequencing, and reverse sequencing was performed in combination with electrophoresis (**a**); the core technologies and sequencing methods of the three common NGS platforms are different: Illumina platform through bridge amplification (**b**), MGI’s DNBSEQ through rolling circle amplification (**c**), and Ion Torrent through “water in oil” technology (**d**) for high-throughput sequencing; TGS completed sequencing by zero-mode waveguide technology (**e**); and FGS completed sequencing by detecting ion changes (**f**).

First-generation sequencing, also known as Sanger sequencing, was the first technology to be widely used in clinical practice for mutation identification [20]. This review catalogs several key events in Sanger sequencing (Figure 2) [20,21]. Sanger sequencing is based on the dideoxy-chain termination method, which separates fragments of different lengths by fluorescence-labeled dideoxynucleotide triphosphate (ddNTP) to achieve DNA sequencing with single-base resolution (Figure 3a). The Sanger sequencing platform integrates Sanger sequencing with capillary electrophoresis and plays important roles in multiple clinical applications.

Sanger sequencing remains the gold standard [22] for orthogonal validation due to its >99.5% base-calling accuracy [23,24]. Contemporary applications extend beyond conventional genotyping to include quality control of CRISPR-Cas9 gene editing, where it detects off-target effects at 0.1% sensitivity [25] and structural validation of extrachromosomal DNA breakpoints in malignancies [26]. Recent engineering advances have mitigated throughput constraints; automated microfluidic systems exemplified by Thermo Fisher’s SeqStudio Flex platform now process 350 samples per run with a 12 h turnaround, tripling efficiency while preserving subclonal detection thresholds at 20% variant allele frequency. Nevertheless, this technology remains constrained by its inability to resolve tumor heterogeneity below this sensitivity ceiling. This challenge may be mitigated through pre-enrichment strategies such as microdissection or digital droplet PCR.

### 3.2. Next-Generation Sequencing (NGS)

Next-generation sequencing (NGS) has transformed molecular diagnostics by enabling massively parallel sequencing of fragmented DNA templates [27]. Major clinical short-read platforms include Illumina, MGI (DNBSEQ), and Ion Torrent, each employing distinct chemistries and library strategies [28]. Illumina technology uses bridge amplification and sequencing by synthesis with reversible terminators for optical base calling; MGI’s DNBSEQ produces DNA nanoballs via rolling circle amplification on patterned arrays to achieve high-density sequencing with reduced PCR bias; Ion Torrent detects hydrogen ions released during nucleotide incorporation and translates pH changes into electrical signals (Figure 3b–d) [29].

Since the introduction of early pyrosequencing instruments and the first human whole-genome sequences, NGS’s throughput and cost efficiency have increased exponentially [30,31]. Large NGS-derived resources (e.g., TCGA, COSMIC) have accelerated both translational research and clinical implementation [32,33]. Compared with first-generation methods, NGS offers substantially higher throughput (hundreds of millions to billions of reads per run), faster turnaround, and a markedly lower cost per base, enabling broad applications from targeted panels and whole-exome sequencing to RNA-seq and metagenomics. The integration of machine-learning methods into variant calling and annotation pipelines has further improved sensitivity and specificity while streamlining reporting workflows, though these algorithms require rigorous clinical validation before deployment.

Key advantages over first-generation sequencing include rapid turnaround, orders-of-magnitude lower cost per base, and billions of reads per run on leading platforms, enabling applications from metagenomics to transcriptomics [34]. Recent AI integration automates variant calling, improving accuracy while significantly shortening the reporting cycle [35]. Despite revolutionizing high-throughput profiling, NGS confronts inherent limitations rooted in its fundamental architecture. The technology’s short-read length (50–300 bp) precludes the comprehensive resolution of structural variants exceeding 1 kb [36]. For genomic regions containing highly repetitive sequences, long fragments, or complex gene fusions, NGS may miss reads or misassemble complex fusions, resulting in false positive or false negative results. NGS cannot directly detect epigenetic modifications such as DNA methylation and can only be implemented after experimental design such as Phosphite treatment of DNA of MeDIP-Seq [37], and the error rates vary from 0.087% to 0.613% [38].

### 3.3. Third-Generation Sequencing (TGS) and Fourth-Generation Sequencing (FGS)

TGS, also known as long-read sequencing (LRS), represented by PacBio HiFi [39], and FGS, represented by Oxford Nanopore technologies (ONT), both deliver transformative capabilities through single-molecule real-time analysis [40,41]. PacBio HiFi achieves >99.9% single-molecule accuracy (Q30+) through circular consensus sequencing (CCS), which generates multiple passes over the same DNA molecule to yield a highly accurate consensus read, clinically validated for glioma classification and molecular subtyping [42,43,44]. Oxford Nanopore’s Q20+ duplex chemistry generates >150 kb reads based on ion current changes [45], enabling ultra-rapid tumor diagnosis and intraoperative clinical applications (Figure 3e) [46]. Historically, PacBio RS systems pioneered single-molecule real-time (SMRT) sequencing using zero-mode waveguide (ZMW) technology, fluorescently labeled nucleotides, and real-time DNA polymerase activity detection (Figure 3f) [41,47,48]. Today, clinical-grade performance is achieved with PacBio HiFi circular CCS reads, which provide Q30+ (>99.9%) accuracy and form the basis for current molecular diagnostics.

TGS and FGS platforms fundamentally circumvent PCR amplification requirements and the associated GC content bias inherent in short-read methods, enabling the direct detection of epigenetic modifications (e.g., 5-methylcytosine), complex structural variants (>50 bp), and phased haplotypes [49]. Early single-pass raw reads from SMRT sequencing exhibited ~10–15% per-base error [50], but current CCS/HiFi workflows convert these signals into Q30+ consensus reads (>99.9% accuracy). Likewise, the ONT duplex mode improves single-read accuracy from ~5–10% raw error to Q20+ consensus (>99%) [51]. Remaining challenges include homopolymer length resolution and a lower throughput compared with NGS [52,53].

## 4. Clinical Applications of Sequencing Technology

**Figure 4:**
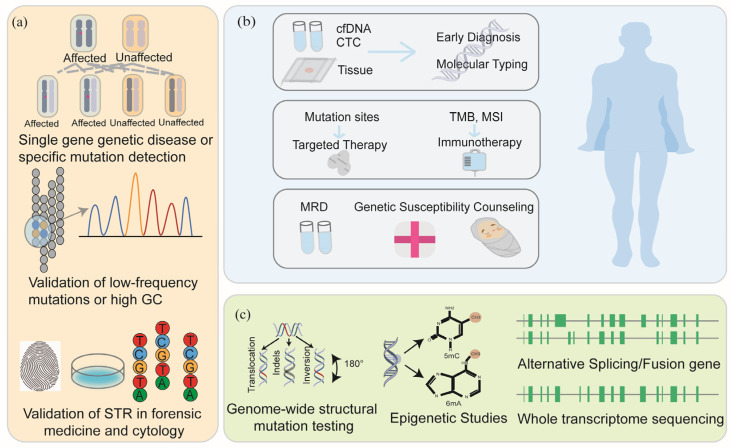
The applications of sequencing technology. Sanger sequencing usually plays a role in single-gene detection and validation, low-frequency mutation and high GC region validation, forensic medicine, and cytology STR validation (**a**); NGS plays an important role in early screening, molecular typing, medication guidance, and prognosis detection of tumors (**b**); TGS and FGS play an important role in whole-genome sequencing, epigenetics, and transcriptome sequencing (**c**).

Sequencing technology plays an important role in clinical practice by accurately detecting individual information at the level of DNA, RNA, and epigenetics, etc. This chapter is divided into several sections according to different sequencing technologies and summarizes the role of sequencing technology in solid tumors (Figure 4).

### 4.1. Applications of Sanger Sequencing in Solid Tumors

Sanger sequencing maintains a critical position in tumor mutation gene and pharmacogenomics (PGXs) detection, forensic identification, and infectious disease surveillance (Figure 4a) [54]. In solid tumor diagnostics, Sanger sequencing fulfills three essential functions [55]. First, it serves as a guideline-mandated validation tool endorsed by organizations including NCCN and the Chinese Society of Clinical Oncology (CSCO), confirming ambiguous results from NGS or PCR assays such as those occurring in GC-rich regions or low-confidence variants [55]. The confirmation of *KIT* and *PDGFRA* mutations by Sanger sequencing to guide TKI therapy is a well-established example [56]. Second, Sanger sequencing represents the established gold standard for microsatellite instability (MSI) analysis. Microsatellite instability high (MSI-H) tumors develop from DNA mismatch repair deficiency (dMMR), resulting in hypermutated genomes with elevated immunogenicity [57]. This phenotype correlates with an enhanced response to PD-1/PD-L1 inhibitors [58,59]. Technically, capillary electrophoresis–based MSI testing using the NCI-recommended ‘2B3D’ panel featuring two mononucleotide repeats (BAT-25 and BAT-26) and three dinucleotide repeats (D5S346, D2S123, and D17S250) balances sensitivity and specificity for solid tumors such as colorectal and endometrial cancers [60]. Third, it facilitates methylation profiling through bisulfite conversion-coupled sequencing to detect clinically relevant 5-methylcytosine patterns in promoter regions [61,62].

Notwithstanding these utilities, Sanger’s analytical sensitivity is limited in routine settings: typical detection limits approximate ~15–20% variant allele frequency (VAF), and lower-frequency variants are more reliably detected using orthogonal high-sensitivity assays such as digital PCRs or deep NGS. Nevertheless, when applied to well-defined validation targets and recurrent hotspot alterations, Sanger remains an efficient, cost-effective tool compatible with clinical workflows.

### 4.2. Applications of NGS in Solid Tumors

Solid tumors necessitate earlier detection during their asymptomatic phase, surpassing the capabilities of traditional pathological examination. While fluorescence in situ hybridization (FISH), comparative genomic hybridization (CGH), and gene expression profiling historically classify challenging tumors [63], advances in sequencing technology now offer novel molecular diagnostic frameworks [64]. The consolidation of NGS into oncology guidelines issued by the NCCN, American Society of Clinical Oncology (ASCO) [15,65], the European Society of Medical Oncology (ESMO) [66], and the CSCO reflects its pivotal role in precision oncology. Regulatory approvals by the FDA and NMPA since 2017 underscore its clinical utility, particularly for large-panel assays exceeding 500 genes (Table 2). This review summarized the clinical application of NGS (Figure 4b) in solid tumors, and we delineated three evidence-based application domains.

#### 4.2.1. Hereditary Tumor Assessment via Integrated NGS Profiling

Hereditary cancers (including breast cancer, prostate cancer, colorectal cancer, etc.) are mainly related to the occurrence of germline mutations. The implementation of NGS multi-gene panels fundamentally transforms hereditary cancer risk management by enabling the comprehensive detection of clinically actionable germline mutations [67,68]. This approach identifies pathogenic variants in high-penetrance susceptibility genes like *BRCA1/2* [69], *TP53* [70], *APC* [71] and emerging markers including RAD51C/D and PALB2. Multiple guidelines indicate that high-risk individuals and families benefit from precise risk stratification, including *BRCA1/2* carriers initiating breast MRI screening at age 25, and prophylactic salpingo-oophorectomy reduces ovarian cancer mortality in BRCA+ individuals [72]. Cascade testing of relatives enables evidence-based interventions with tamoxifen chemoprevention reducing breast cancer risk by 53% in *PALB2* carriers [73,74], while risk-reducing salpingo-oophorectomy decreases ovarian cancer mortality [75]. Clinical implementation reduces hereditary cancer mortality by 40% within 10 years compared to standard care through early detection and preventive strategies. Contemporary guidelines mandate germline NGS testing for *BRCA1/2* in breast cancer patients regardless of age based on the 5.1% mutation prevalence demonstrated in patients over 70 years [76]. The CSCO emphasizes the detection of *BRCA1/2* germline mutations in populations at high risk for breast and ovarian cancer to guide the use of *PARP* inhibitors.

#### 4.2.2. NGS-Driven Paradigm Shift in Early Cancer Detection

The convergence of NGS, artificial intelligence (AI), and multi-omics profiling has fundamentally transformed early cancer detection. Liquid biopsy technologies now enable non-invasive identification of tumor-derived signals including DNA methylation [77,78], somatic mutations, fragmentomics patterns, and protein biomarkers with unprecedented sensitivity and specificity. This paradigm shift addresses critical limitations of traditional screening methods, such as invasive procedures, low positive predictive values (PPVs), and organ-specific constraints.

NGS-driven liquid biopsies achieve a single-CpG resolution for methylation analysis, detecting hypermethylated promoters (e.g., SEPT9, NDRG4) [79]. As the first global NGS-based liquid biopsy for early colorectal cancer (CRC) detection approved by the FDA in 2024, the Shield assay demonstrates 93.9% sensitivity for CRC and 90.6% specificity for advanced neoplasia [80]. It achieves significantly higher detection rates for CRC and advanced precancerous lesions compared to fecal immunochemical testing (FIT). Galleri, the world’s first solid tumor early screening kit based on NGS and blood biopsy technology, is aimed at DNA methylation detection. These performance metrics align with current guideline recommended non-invasive screening methods, which show aggregate CRC sensitivities ranging from 74% to 92% [81].

Contemporary molecular taxonomy through NGS supersedes the traditional histopathological classification of solid tumors by integrating multi-omics signatures to delineate tumors with divergent clinical behaviors despite morphological similarities [82,83,84]. Conventional pathological classification yields diagnostic discordance in 29% of sarcomas [85,86], whereas NGS achieves a 10.5–26.3% reclassification of cases [79,87], substantially enhancing therapeutic precision through the identification of druggable targets like MDM2 and CDK4 amplifications [88,89]. The strong auxiliary effect of NGS for this type of solid tumor has been clearly mentioned in the guidelines. NCCN and ESMO clearly recommend NGS testing for advanced or recurrent/metastatic thyroid cancer [66,90], soft tissue sarcoma [91,92], and osteosarcoma [93,94]. NCCN lists *BRAF* and *RET* as mandatory testing genes for thyroid cancer and emphasizes the necessity of fusion gene testing in the diagnosis of sarcoma [90]. In addition, for cancers of unknown primary (CUP), NGS reclassifies 55% of cases by revealing clinically actionable biomarkers such as MSI-H status, tumor mutational burden–high (TMB-H), and pathogenic germline variants in homologous recombination repair genes [95].

#### 4.2.3. Applications of NGS in Precision Oncology

NGS underpins precision oncology by enabling more accurate targeting of treatment and prognostic stratification across solid malignancies [96]. First, in NSCLC, NGS identifies driver mutations (e.g., *EGFR*, *ALK*, and *ROS1*) that guide tyrosine kinase inhibitor (TKI) therapy, yielding substantially improved progression-free survival relative to conventional chemotherapy [97]. Beyond lung cancer, targeting homologous pathogenic variants—such as *BRAF* V600E across tumor types—or *HER2* overexpression in diverse cancers demonstrates the tissue-agnostic potential of molecular profiling [98,99]. Second, NGS reveals co-mutation patterns and resistance mechanisms that modulate therapeutic response [100,101]. For example, concurrent *TP53* mutation in the *EGFR*-mutant NSCLC diminishes efficacy of osimertinib [102,103,104]. Serial monitoring of circulating tumor DNA (ctDNA) permits the early detection of resistance (e.g., emergence of the *EGFR* T790M mutation), facilitating timely therapeutic switching [105,106,107]. In gastrointestinal stromal tumors, rare primary resistance mutations (such as *PDGFRA* D842V) identified via NGS have led to the development of subtype-specific agents (e.g., avapritinib) with high response rates in genetically defined patient cohorts [108,109]. This study integrates three multicenter phase III drug trials in advanced NSCLC (Table 3). For advanced/metastatic NSCLC, NGS can provide technical support for clinical trial drug efficacy analysis by accurately detecting rare mutations.

Third, in the immuno-oncology realm, NGS supplies predictive biomarkers (TMB, MSI [35,58,113]) and deciphers immune evasion mechanisms (e.g., *B2M* mutations, *JAK1/2* truncations [114,115]). The design of tumor vaccines based on neoantigens encoded by mutated genes has emerged as an emerging component of immunotherapy [116,117,118]. Accurate identification of tumor-specific mutations by NGS, combined with the assessment of antigen immunogenicity by bioinformatics tools, enables the timely and low-cost identification of personalized neoantigens [116]. Moreover, antigen-discovery applications—such as neoantigen vaccine design [116,119,120] and T-cell receptor (TCR) [121,122] repertoire analysis—are increasingly informed by NGS data. These approaches support personalized immunotherapeutic strategies and therapeutic monitoring (ctDNA, cytokine dynamics).

Finally, for prognostic stratification, NGS-informed molecular staging is supplanting purely anatomical staging in several tumor types. Key biomarkers include TMB and MSI status with 99.7% concordance versus immunohistochemistry, facilitating precision immunotherapy selection [123]. Pan-cancer data confirm significantly elevated ORR (29% vs. 6%) in TMB-H versus low cohorts [124,125]. Prospective trials validate molecular staging systems integrating *TERT* mutations, *CDKN2A* deletion, and 1p/19q co-deletion, outperforming traditional staging in glioma prognosis [126].

Despite its transformative clinical value, short-read NGS inherently struggles to capture long repetitive or homologous genomic regions, GC-rich loci, and structural rearrangements exceeding its read length constraints [127]. These sequencing blind spots can obscure clinically significant variants involved in tumor initiation or predisposition [128]. A striking example is the synthetic transcription elongation factors described by Erwin et al., which demonstrated that repetitive heterochromatin can physically halt productive transcription elongation and require specialized mechanisms to resume elongation [129]. This observation underscores how incomplete coverage of repetitive regions—even in non-coding, regulatory, or intergenic loci—may obscure biologically relevant transcriptional and mutational signals, particularly those associated with early tumor evolution. Such incomplete sequencing limits the accuracy of molecular warning systems, particularly in liquid biopsy-based early detection assays, where tumor-derived signals may be underestimated [127]. These gaps underscore the need for integrative validation strategies and the progressive adoption of long-read platforms capable of spanning complex genomic regions to ensure comprehensive variant discovery in precision oncology [130].

Collectively, these applications illustrate how NGS not only guides treatment selection but also reshapes prognosis and trial design in solid tumors.

### 4.3. Applications of TGS and FGS in Solid Tumors

TGS resolves complex genomic landscapes with unprecedented accuracy, while FGS enables ultra-long reads and direct epigenomic profiling (Figure 4c). TGS also improves diagnostic yield in hereditary disorders [131]. The accurate genome assembly and ultra-long read length make TGS and FGS play a significant advantage in single nucleotide variation, structural variation, and CpG methylation molecules in tumors including breast cancer [132,133,134]. Complex regions of the genome that cannot be covered by NGS, such as large copy number amplification, chromosomal translocations, and repetitive sequence functional elements (such as Alu sequences), can be analyzed by TGS to generate high-quality genome maps [47,135]. These structural variants may be important tumor markers or driver genes, such as the large fragment deletion of the *LRP1B* gene and chromosome 10/16 translocation variants found in several tumors, which are directly related to the degree of tumor malignancy and the selection of therapeutic targets [136]. Simultaneously, FGS excels in complex infection scenarios for the Ebola virus [137], infectious endocarditis [138], influenza [139], pertussis [140], and meningitis [141].

FGS’s long reads resolve previously “unmappable” genomic regions. In thalassemia diagnostics, the Comprehensive Analysis of Thalassemia Alleles (CATSA) framework achieved 100% accuracy for rare variants like the α3.7III subtype by spanning GC-rich HBA1/HBA2 loci [142]. For hemophilia A, TGS demonstrates extremely high sensitivity for detecting all classes of F8 variants, significantly enhancing genetic screening and molecular diagnosis [143]. The convergence of TGS with spatial barcoding technologies (e.g., 10x Genomics Xenium) enables the three-dimensional reconstruction of clonal evolution trajectories within tumor niches [144]. In pancreatic ductal adenocarcinoma, integrated analysis of *KRAS* G12D allele-specific methylation and stromal fibroblast interactions predicts early metastasis [145,146].

The convergence of FGS with AI and spatial biology will unlock new clinical dimensions. AI models like AI-MARRVEL [147] and GeneT [148] now predict antibiotic resistance phenotypes directly from raw nanopore signals while bypassing bioinformatic pipelines. Crucially, digital twinning initiatives such as SimBioSys TumorScope simulate tumor dynamics using FGS-derived structural variant profiles and epigenetic states, predicting the response to polytherapy regimens with >90% accuracy in breast cancer trials [149]. As these technologies mature, FGS will transition from a diagnostic tool to the core engine of adaptive cancer therapy, one capable of recalibrating treatment in sync with the evolving genomic landscape of malignancy.

## 5. Convergent Futures of Tumor Sequencing Platforms

The rapid development of sequencing is ushering in a new era of individualized precision treatment for solid tumors. By comprehensively decoding cancer driver mutations, molecular subtypes, and dynamic evolution, sequencing technology provides an unprecedented core driving force for clinically accurate diagnosis, individualized targeting and immunotherapy regimens, and real-time analysis of drug resistance mechanisms. With continued improvements in depth, throughput, and single-cell/spatial methods, we can build more refined, spatiotemporal tumor maps. How to efficiently integrate and interpret the resulting massive multidimensional omics data and translate them into clinically actionable intervention strategies will be the key proposition leading the precision diagnosis and treatment of solid tumors to a new height and also the most promising and challenging development direction in the future.

### 5.1. Emerging Biological and Analytical Frontiers

The precision oncology revolution, while propelled by sequencing advancements, now confronts several biological and operational frontiers beyond current technical solutions. Intratumoral epigenetic plasticity manifests as dynamically shifting methylation landscapes that evade single-timepoint assays clinically evidenced by MGMT promoter reversion in 37% of recurrent glioblastoma patients [150]. This epigenetic state oscillation circumvents temozolomide cytotoxicity despite baseline promoter methylation positivity. Mitochondrial genome heterogeneity remains largely unexplored [151]. Current single-cell mtDNA sequencing faces systemic limitations including low on-target efficiency (~20%) [152], unavoidable nuclear mitochondrial DNA segments (NUMTs) contamination, amplification-induced allelic dropout, and prohibitive costs, collectively compromising the reliable quantification of mitochondrial mutation load per cell [153,154]. While extrachromosomal DNA (ecDNA) presented in 17.1% of all tumor samples [155], accurate circRNA quantification via NGS confronts the inherent limitations from template fragmentation-induced back-splice junction loss compounded by ultra-low transcript abundance, resulting in false positive rates exceeding 45%, even with advanced computational correction [156,157]. The long-read nanopore sequencing demonstrated in four seminal 2021 methodologies (isoCirc [158], CIRI-long [159], circNick-LRS [160], and circFL-seq [161]) enables full-length circRNA recovery through RCA-based amplification or enzyme-linearized intermediates, revealing extensive structural diversity including fusion isoforms and internal complexity undetectable by short-read sequencing. However, ultra-low expression levels coupled with intrinsic sequence homology to parental linear transcripts still impose fundamental analytical bottlenecks in sensitive circRNA characterization. These biological blind spots necessitate convergent innovation pathways.

### 5.2. Translational and Operational Challenges in Clinical Implementation

The convergence of sequencing revolutions with solid tumor oncology has irrevocably transformed cancer diagnostics. While NGS-enabled ctDNA profiling and tissue molecular stratification now guide targeted therapies, we stand at a pivotal inflection point where NGS unravels chromosomal instability epiphenomena. Yet this technological ascendance intensifies core paradoxes as urgent clinical decisions await comprehensive genomic signatures even while petabytes of tumor data overwhelm interpretation pipelines. Competitive innovation accelerates this transformation. Integrating Oxford’s intraoperative methylation tracking, BGI’s CTC enumeration, GenapSys’ portable *BRCA* testing, and Genia’s microenvironment mapping now constitutes solid oncology’s foundational sensing infrastructure. However, the future demands not merely faster sequencers but integrated frameworks where temporal, spatial, and functional tumor data coalesce into dynamic therapeutic roadmaps.

In this new epoch, sequencing ceases being a mere tool and becomes the foundational architecture through which we re-engineer cancer precision medicine. Given the ethical considerations, clinical sequencing should be governed by robust safeguards.

### 5.3. Ethical and Global Considerations in Precision Oncology

While AI offers transformative potential in oncology diagnostics, it also introduces an array of ethical challenges. Algorithmic bias—caused by the misrepresentation of specific populations during training—can reinforce existing inequities, leading to misdiagnoses or inadequate care, especially among racial and gender minorities [162]. Additionally, issues surrounding accountability and moral responsibility—sometimes referred to as “moral outsourcing”—arise when the blame is shifted away from developers and toward the technology itself. Moreover, differential performance across demographic groups can erode patient trust and exacerbate disparities in treatment access. Tackling these challenges will require both technical solutions—such as fairness-aware models and federated learning—and governance measures, including robust consent mechanisms, equity-focused policy frameworks, and sustained oversight across the AI lifecycle.

Although NGS has become integral to precision oncology in high-income countries, its adoption remains uneven across healthcare systems [163]. Key barriers include limited funding and infrastructural support, shortages of trained personnel, and supply chain constraints, which disproportionately affect low-resource settings [164,165]. These disparities not only hinder access to genomic testing but also limit the diversity of genomic datasets, which exacerbate bias in machine-learning models and reduce diagnostic accuracy for underrepresented populations [166]. To overcome these obstacles, coordinated international efforts—including technical assistance, cross-border collaboration, and tailored capacity-building programs—are essential to ensure equitable access to advanced sequencing technologies.

## 6. Conclusions

Sequencing technologies have revolutionized the landscape of solid tumor research and clinical practice by enabling the precise delineation of genetic alterations, molecular subtypes, and tumor evolution. This review summarizes the trajectory from early Sanger sequencing to next-generation and long-read platforms, highlighting how each technological advancement has deepened our understanding of tumorigenesis and transformed clinical management. By integrating laboratory-based mutation profiling with evidence from the literature, we provide a comprehensive framework linking molecular discoveries to clinical translation, underscoring the central role of sequencing as the operational backbone of precision oncology.

Looking forward, the continued refinement of sequencing platforms, integration of multi-omics layers, and development of real-time spatiotemporal tumor monitoring could enable the construction of dynamic and individualized therapeutic roadmaps. The key challenge will be harmonizing massive, heterogeneous datasets into clinically actionable insights while maintaining cost-effectiveness, interpretability, and ethical oversight. Success will require close collaboration between clinicians, bioinformaticians, and technology developers, ultimately transforming sequencing from a diagnostic tool into the foundational infrastructure that guides adaptive cancer care.

## Figures and Tables

**Table 1:** The 35 most common solid tumors and their common mutation genes.

Origin of Tumor	Type of Tumor	Common Mutation Genes
Epithelial origin	Female breast	*TP53,* *ERBB2, BRCA1/2, PIK3CA/AKT1/PTEN, ESR1, TROP-2*
	Lung cancer	*EGFR, ALK, ROS1, BRAF, NTRK, KRAS, TP53, MET, RET, ERBB2, NRG1*
	Prostate	*TMPRSS2-ERG, SPOP, PTEN, TP53, AR, BRCA1/2, CDK12, MSH2/6*
	Non-melanoma of skin	*TP53, PTCH1 and SMO,* *SUFU,* *RAS, NOTCH, FAT1*
	Colon	*APC, KRAS, NRAS, TP53, PIK3CA, SMAD4, CTNNB1, BRAF, ERBB2, RET, POLE, POLD1, NTRK1, NTRK2, NTRK3.*
	Stomach	*TP53, CDH1, KRAS, PIK3CA,* *ERBB2, ARID1A*
	Liver	*TP53, TERT, CTNNB1, AXIN1, ARID1A, RB1, TSC2*
	Rectum	*APC, KRAS, NRAS, TP53, PIK3CA, SMAD4, BRAF, ERBB2, RET, POLE, POLD1, NTRK1, NTRK2, NTRK3*
	Esophagus	*TP53, PIK3CA, FBXW7, KRAS, CDKN2A, NFE2L2, ZNF750, NOTCH1*
	Bladder	*FGFR3, TP53, RB1, PIK3CA, KDM6A*
	Pancreas	*KRAS, TP53, CDKN2A, SMAD4, BRCA1/2, MLL3, PALB2, ATM, ARID1A*
	Kidney	*VHL, PBRM1, MET, ERBB2,* (Clear cell renal cell carcinoma) *CDKN2A, CDKN2B, CTNNB1,* (Papillary renal cell carcinoma) *TERT, BAP1,* (Chromophobe cell carcinoma) *KDM5C, TERT, BAP1, TP53,* (Collecting duct carcinoma)
	Corpus uteri	*ARID1A, PTEN, MUC16, PIK3CA, POLE, MMR, TP53, ERBB2, BRCA1/2*
	Lip, oral cavity	*TP53, CDKN2A, PIK3CA, PIK3CA, HRAS, NOTCH1*
	Melanoma of skin	*BRAF, KRAS/NRAS, KIT, KAT,* *PIK3CG*
	Larynx	*TP53,* *CDKN2A, PIK3CA, NOTCH1, FAT1, CCND1,* *LAMA3*
	Nasopharynx	*LMP1/2, TP53, PIK3CA, CDKN2A, IKK*
	Gallbladder	*KRAS, TP53, CDKN2A, PIK3CA, ARID1A, FGFR2,* *ERBB2, BAP1, IDH1/2, MLL3/KMT2C*
	Oropharynx	*HPV, PIK3CA, FAT1, CDKN2A, TP53, NOTCH1, CASP8, SOX2*
	Hypopharynx	*TP53, PIK3CA, CDKN2A, HRAS, NOTCH1*
	Salivary glands	*MYB-NFIB, RET, NR4A3, NBN*
	Anus	*PIK3CA, MLL2/3, TP53, ATM, HUWE1, BRCA1/2, EP300, SMARCB1, SMARCA4*
	Vulva	*HPV, PIK3CA, TP53, KIT, NF1*
	Penis	*TP53, PIK3CA, CDKN2A, HRAS*
	Mesothelioma	*BAP1, CDKN2A, NF2, TP53, RB1, DDR2, FGFR, SEDT2*
	Vagina	*TP53, PIK3CA, CDKN2A, HRAS*
Mesenchymal origin	Kaposi sarcoma	*HHV-8, PIK3CA, TP53, RAC1, CCNB1, VEGF, HIF1A*
Nervous system origin	Brian, nervous system	*IDH1/2, ATRX, TERT, MGMT, EGFR,* (Glioma) *PTCH1, SMO, TP53, MYCN,* (Medulloblastoma) *NF2, LZTR1,* (Neurilemmoma) *NF2, TRAF7, KLF4, SMO,* (Meningiomas)
Germ cell origin	Ovary	*BRCA1/2, TP53, KRAS, PIK3CA, PTEN, ARID1A*
	Testis	*KIT, KRAS, TP53, CTNNB1*
Thyroid origin	Thyroid	*BRAF, RAS, RET, TERT, TP53, ALK, PAX8*
Others	Cervix uteri	*HPV, TP53, KRAS, PIK3CA, PTEN, CCND1, FGFR, NOTCH, MLL, PAX*

**Table 2:** Information on the eight large-scale NGS panel kits approved by the FDA/NMPA for marketing.

Test Kit Name	Company	Year of Approval	Number of Genes	Sequencing Instrument	Approved Use
MSK-Impact	MSK	2017	468	HiSeq 2500, Illumina	Tumor qualitative IVD detection products (including MSI)
FoundationOne CDX	Foundation Medicine	2017	324	HiSeq 4000, Illumina	Companion diagnosis: mutation, fusion, TMB, and MSI
PGDx elio tissue complete	PGDx	2020	505	NextSeq 550DX, Illumina	Tumor qualitative IVD detection products (including MSI and TMB)
FoundationOne Liquid CDX	Foundation Medicine	2020	324	NextSeq 6000, Illumina	Companion diagnosis: mutation and fusion
NYU Langone Genome PACT	NYU Langone Health (NYU)	2021	607	NextSeq 500/550, Illumina	Tumor qualitative IVD detection products: Point mutations and insertions or deletions of less than 35 bp
xT CDx	Tempus Labs, Inc. (Tempus)	2023	648	NovaSeq 6000, Illumina	Companion diagnostics: mutation; IVD: MSI
GENESEEQPRIME™ TMB	Geneseeq	2023	425	NextSeq 550DX/HiSeq 4000, Illumina	TMB in EGFR mutation-negative and ALK-negative non-squamous NSCLC patients
TruSight Oncology Comprehensive	Illumina	2024	517	NextSeq 550DX, Illumina	Companion diagnostics: fusion, tumor qualitative IVD detection products (including mutation and TMB)

**Table 3:** Clinical trials of drugs for non-small-cell lung cancer.

Medication Regimen	Mutation Genes	Prognostic Measures	Data Resources
Dato-DXd	*EGFR* 19Del, L858R, and T790M	ORR:43% (95%CI: 34–52%), median DOR: 7.0 months (95% CI: 4.2–9.8), median PFS: 5.8 months (95% CI: 5.4–8.2), and median OS: 15.6 months (95% CI: 13.1–19.0)	[110]
sac-TMT vs. docetaxel	*EGFR* mutation with inhibitors resistant to tyrosine kinase	ORR: 29% better than docetaxel (95% CI:15–43%), median PFS: 6.9 v 2.8 months (HR 0.30, 95% CI 0.20–0.46), and 1 year’s OS rate: 73% v 54% (HR: 0.49, 95% CI: 0.27–0.88)	[111]
alectinib with bevacizumab	*ALK* rearrangement	1 year’s PFS rate: 97.1% (95% CI: 92.6–100%), 36 months’ PFS rate: 64.2% (95% CI: 56.1–85.2), and 36 months’ OS rate: 87.9% (95% CI74 ~96.6)	[112]

## Data Availability

The data presented in this study are available on request from the corresponding author upon request. We are more than willing to share our original data with readers who are interested in our research. However, considering that the clinical data contain sensitive information such as the patient’s name, hospitalization number, etc., in order to protect the patient’s privacy, we are unable to disclose all the original patient data.
